# Identification of *NRAS* Downstream Genes with CRISPR Activation Screening

**DOI:** 10.3390/biology11111551

**Published:** 2022-10-23

**Authors:** Akiya Tatsumi, Haruka Hirakochi, Satomi Inoue, Yosuke Tanaka, Hidehiro Furuno, Masumi Ikeda, Sachiko Ishibashi, Towako Taguchi, Kouhei Yamamoto, Iichiroh Onishi, Zohar Sachs, David A. Largaespada, Masanobu Kitagawa, Morito Kurata

**Affiliations:** 1 Department of Comprehensive Pathology, Graduate School of Medical and Dental Sciences, Tokyo Medical and Dental University, Bunkyo-ku, Tokyo 113-8510, Japan; 2 Department of Molecular Pathology, Graduate School of Medical and Dental Sciences, Tokyo Medical and Dental University, Bunkyo-ku, Tokyo 113-8510, Japan; 3 Department of Medical Technology & Sciences, School of Health Sciences at Narita, International University of Health and Welfare, Narita, Chiba 286-8686, Japan; 4 Masonic Cancer Center, University of Minnesota, Minneapolis, MN 55455, USA; 5 Division of Hematology, Oncology, and Transplantation, Department of Medicine, University of Minnesota, Minneapolis, MN 55455, USA

**Keywords:** CRISPR screening, NRAS

## Abstract

**Simple Summary:**

*NRAS* mutations constitutively activate cell proliferation signaling in malignant tumors. The elucidation of *NRAS* downstream signaling genes may lead to the control of *NRAS*-mutant tumors. Genome-wide CRISPR activation screening was performed on THP-1 B11, in which *NRAS* expression and cell proliferation could be regulated by doxycycline. Multiple candidate genes were identified from clones that survived in *NRAS*-off, and three genes—*DOHH*, *HIST1H2AC*, and *TAF6*—were finally identified as being downstream of *NRAS* signaling and confirmed to contribute to the proliferation of THP-1 cells. These molecules are promising new therapeutic targets for *NRAS*-mutant leukemia.

**Abstract:**

Mutations in *NRAS* constitutively activate cell proliferation signaling in malignant neoplasms, such as leukemia and melanoma, and the clarification of comprehensive downstream genes of *NRAS* might lead to the control of cell-proliferative signals of *NRAS*-driven cancers. We previously established that *NRAS* expression and proliferative activity can be controlled with doxycycline and named as THP-1 B11. Using a CRISPR activation library on THP-1 B11 cells with the *NRAS*-off state, survival clones were harvested, and 21 candidate genes were identified. By inducting each candidate guide RNA with the CRISPR activation system, *DOHH*, *HIST1H2AC*, *KRT32*, and *TAF6* showed higher cell-proliferative activity. The expression of *DOHH*, *HIST1H2AC,* and *TAF6* was definitely upregulated with *NRAS* expression. Furthermore, MEK inhibitors resulted in the decreased expression of DOHH, HIST1H2AC, and TAF6 proteins in parental THP-1 cells. The knockdown of *DOHH*, *HIST1H2AC*, and *TAF6* was found to reduce proliferation in THP-1 cells, indicating that they are involved in the downstream proliferation of *NRAS*. These molecules are expected to be new therapeutic targets for *NRAS*-mutant leukemia cells.

## 1. Introduction

RAS is a low-molecular-weight GTP-binding protein, and its active form stimulates downstream kinase cascades, leading to various cellular responses, such as cell proliferation and apoptosis [1,2]. Of the three RAS isoforms (KRAS, NRAS, and HRAS), activated *NRAS* mutations are common in melanoma (17%) and hematological malignancy (9.6–19%) [3]. In recent years, KRAS^G12C^ inhibitors (AMG510) have been developed and approved for *KRAS^G12C^*-mutated non-small cell lung cancer (NSCLC) patients [4]; however, there is no approved drug for *NRAS*-mutant tumors [5]. The inhibition of the downstream factors of *NRAS* has been proposed as an alternative therapeutic strategy for *NRAS*-mutant tumors [6,7]; however, the inhibition of known downstream molecules, such as MEK alone, has limited therapeutic efficacy [8]. Therefore, the current study aims to comprehensively identify *NRAS* downstream genes as new candidate molecules for the therapeutic targeting of *NRAS*-mutant tumors.

CRISPR screening identifies the responsible genes by focusing on phenotypic changes that are manifested by inducing a random gene knockout or activation using a guide RNA (gRNA) library that targets numerous genes [9,10]. This technology enables the comprehensive identification of novel genes related to various events, such as drug resistance genes, regulatory genes, and synthetic lethal genes. In the present study, CRISPR activation screening was applied to THP-1 B11 cells whose *NRAS* expression could be regulated by doxycycline (Dox). The cells were generated by knocking out endogenous *NRAS* with CRISPR/Cas9 and introducing an exogenous Dox-regulated *NRAS^G12V^* expression vector into leukemia cells carrying the *NRAS^G12D^* mutation and whose growth depended on the *NRAS* signaling pathway [11]. CRISPR activation screening was applied to THP-1 B11 cells that were addicted to *NRAS^G12V^* to identify genes that could replace the *NRAS^G12V^*-addicted genes. An analysis of the activated genes is expected to identify novel *NRAS* downstream genes that can substitute for the *NRAS* function.

## 2. Materials and Methods

### 2.1. Cell Culture

The THP-1-derived B11 cell line was generated by an endogenous *NRAS* knockout with CRISPR/Cas9 and transduction with an exogenous Dox-inducible *NRAS* expression vector, as previously described [11]. HEK293Ts (human embryonic kidney cells 293) were purchased from the RIKEN cell bank (Riken Cell Bank, Tsukuba, Japan). MIA PaCa-2 (human pancreatic cancer cell line) and HL-60 (Human promyelocytic leukemia cell line) were obtained from the JCRB Cell Bank (National Institutes of Biomedical Innovation, Health and Nutrition, Osaka, Japan). The B11 cells were cultured in RPMI-1640 medium (FUJIFILM Wako Pure Chemical Corporation, Osaka, Japan) and supplemented with 1 µg/mL of Dox (Sigma-Aldrich, St. Louis, MO, USA); Dox was removed as needed. The THP-1 and HL-60 cells were maintained in RPMI-1640 medium. The HEK293T cells were cultured in DMEM medium (FUJIFILM Wako Pure Chemical Corporation) and the MIA PaCa-2 cells were cultured in EMEM medium (FUJIFILM Wako Pure Chemical Corporation). The THP-1, HEK293T, and MIA PaCa-2 cell lines were cultured in a medium supplemented with 10% fetal bovine serum (Thermo Fisher Scientific, Waltham, MA, USA) and 1% penicillin/streptomycin (FUJIFILM Wako Pure Chemical Corporation). The HL-60 cell line was cultured in a medium supplemented with 20% fetal bovine serum and 1% penicillin/streptomycin. All cell lines were maintained at 37 °C with 5% CO_2_.

To evaluate the effect of the Dox-inducible NRAS, THP-1 B11 cells were precultured in the absence of Dox for 5 days, and 1.0 × 10^6^ cells/well in a 6-well plate were either exposed or not exposed to Dox for 72 h. In the experiments with inhibitors, the THP-1 parental cell line and the HL-60 cell line were seeded at 2.0 × 10^6^ cells/well in a 6-well plate and cultured for 48 h with MEK inhibitor (MEK-I) PD184161 (Santa Cruz Biotechnology, Santa Cruz, CA, USA) (5 μM) dissolved in DMSO. The MIA PaCa-2 cell line was treated with 10 µM MEK inhibitor. Cells treated with DMSO only were used as controls.

### 2.2. CRISPR Activation Screening and Plasmid Construction

Lentivirus induction was performed as described previously [12]. The lentivirus plasmids lentiMPHv2 (Addgene, #89308) and lentidCAS-VP64_blast (Addgene, #61425) and the Human CRISPR Activation Library (SAMv1) (Addgene, #1000000074) were used. After the lenti sgRNA(MS2)_zeo backbone (Addgene, #61427) plasmid was linearized by the restriction enzyme BsmBI (Thermo Fisher Scientific), the fragment was isolated from agarose gels and purified using a gel extraction kit (Promega, Madison, WI, USA). The gRNA sequences for the identified candidate genes were referenced from the SAM library list, and two complementary gRNA oligo DNAs were synthesized. The oligo DNAs annealed by T4 polynucleotide kinase (Takara, Osaka, Japan) were individually cloned into the BsmBI-digested gRNA expression cassette using T4 DNA ligase (New England Biolabs, Ipswich, MA, USA).

The THP-1 B11 cell line was infected with lentiMPHv2 and lentidCAS-VP64_blast vector, followed by 200 µg/mL of hygromycin (Invitrogen, Carlsbad, CA, USA) and 10 µg/mL of blasticidin (Nakarai Tesque, Kyoto, Japan) selection for 2 weeks, respectively. The obtained cells (THP-1 B11-MPH-dCas9-VP64 cells) were used in further experiments. The THP-1 B11-MPH-dCas9-VP64 cells were infected with the CRISPR activation library (lentiSAMv1), followed by 300 µg/mL of zeocin (InvivoGen, San Diego, CA, USA) selection for 2 weeks. The THP-1 B11 cells were always cultured under 1 µg/mL of Dox during infection. Cell pools randomly gene-activated with the CRISPR activation library were washed with PBS to remove Dox and were continued to be cultured in a Dox-free medium. Cell colonies grown in the absence of Dox were isolated under a microscope, and each cell clone was established. The genomic DNA of the established cell clones was extracted with a previously reported method using ProK [12]. Regions containing gRNAs integrated into genomic DNA were PCR-amplified with GoTaq^®^ Master Mix (Promega) with the following primers: forward, 5′-GAGGGCCTATTTCCCATGAT-3′; reverse, 5′-GGAGCCAGTACACGACATCA-3′. The PCR product was ligated to pGEM^®^-T Easy Vector Systems (Promega) by T-A cloning, and then, colony PCR was performed to check for positive clones with the following primers: forward, 5′-GAGGGCCTATTTCCCATGAT-3′; reverse, 5′-TAATACGACTCACTATAGGG-3′. The gRNA sequences were then identified by Sanger sequencing with the same forward primers as above.

After individual gRNA expression plasmids targeting the candidate genes were generated, the plasmids were introduced into THP-1 B11-MPH-dCas9-VP64 cells by infection, followed by 2 weeks of selection with 300 µg/mL of zeocin.

### 2.3. Cell Proliferation

The THP-1 B11 cells were cultured for 4 days without Dox, and then the cells were exposed to Dox. Cells without and with Dox were counted using trypan blue after 3 and 5 days. A cell cycle analysis was performed after 5 days of culture of the cells without and with 1 μg/mL of Dox, per previous reports [13]. Cells stained with 25 μg/mL of propidium iodide (Sigma-Aldrich) were analyzed with BD FACS (Becton Dickinson, San Jose, CA, USA). These experiments were performed in triplicate.

The B11 cells were precultured in a medium without Dox and then seeded with 1.0 × 10^4^ cells for each well of a 96-well plate in media with a step dilution of Dox (0, 0.0195, 0.0391, 0.0781, 0.156, 0.313, 0.625, 1.25, 2.5, 5, and 10 µg/mL). The MTS activity was estimated at 72 h by measuring the 490 nm absorbance at 3 h using CellTiter 96^®^ Aqueous MTS Reagent Powder (Promega) and ELx808 (BioTek/Agilent, Santa Clara, CA, USA). This experiment was performed in quadruplicate.

Cells in which individual candidate genes were activated by CRISPR activation were seeded with 2.5 × 10^4^ cells/well in 96-well plates and subjected to an MTS assay in the same manner as above. This experiment was performed in triplicate. Cells transduced with IL1β-targeted gRNA were used as the controls. The gRNA sequence was 5′-AAAAACAGCGAGGGAGAAAC-3′.

### 2.4. RT-qPCR

A total of 1.0 × 10^5^ cells were collected, and the total RNA was extracted and reverse-transcribed using a ReverTra Ace qPCR RT Master Mix kit (TOYOBO, Osaka, Japan) according to the manufacturer’s protocol. The acquired cDNA was used as a template for quantitative reverse transcription PCR (RT-qPCR). Briefly, cDNA was analyzed on an ABI Prism 7900HT (Applied Biosystems, Foster, CA, USA) using the THUNDERBIRD^®^ Probe qPCR Mix (TOYOBO). The primers used were as follows: *β-actin*, 5′-CACAGAGCCTCGCCTTTGCC-3′ (forward), 5′-CACAGAGCCTCGCCTTTGCC-3′ (reverse); *DOHH*, 5′-GACGGGTCCTGAGTCTCCA-3′ (forward), 5′-GTCACCATCGTGCTGTCAAT-3′ (reverse); *TAF6*, 5′-CTGGACAGGAAGTGCGATG-3′ (forward), 5′-CAGGACCACTCTGACCATCA-3′ (reverse); *KRT32*, 5′-TGGGGACCGCCTTAACATCG-3′ (forward), 5′-ATGGCCTCGTACTGACACCG-3′ (reverse); and *HIST1H2AC*, 5′-GGTAAGCAAGGAGGCAAAGC-3′ (forward), 5′-AGTTGCCTTTACGGAGCAGG-3′ (reverse). The qPCR cycling conditions were as follows: 40 cycles of 95 °C for 15 s and 60 °C for 45 s, followed by a dissociation stage (95 °C for 15 s, 60 °C for 15 s, and 95 °C for 15 s). The relative mRNA expression was analyzed using the delta–delta Ct method and normalized to *β-actin* mRNA levels. All analyses were performed in biological triplicates.

### 2.5. Western Blotting

Western blotting (WB) was performed according to previous reports [13]. The primary antibodies used were anti-β-actin (#4970, Cell Signaling Technology, Danvers, MA, USA), anti-NRAS (#10724-1-AP, Proteintech, Chicago, IL, USA), anti-DOHH (#254866, Abcam, Cambridge, MA, USA), anti-HIST1H2AC (#15953-1-AP, proteintech), anti-TAF6 (#ab76922, Abcam), anti-p44/42 MAPK (Erk1/2) (#4695, Cell Signaling Technology), and anti-Phospho-p44/42 MAPK (Erk1/2) (Thr202/Tyr204) (#9101, Cell Signaling Technology). Each primary antibody was diluted to 1:1000 and incubated overnight. The secondary antibody, diluted to 1:5000, was incubated at room temperature for 1 h. Protein signals were detected using ChemiDoc XRS Plus Systems (Bio-Rad, Hercules, CA, USA) after a 5 min reaction with the ECL substrate (Bio-Rad). Original images of Western blot analysis please see Supplementary File S1.

### 2.6. Small Interfering RNA (siRNA)

Small interfering RNAs (30 pmol/sample) were transfected into THP-1 cells (1.0 × 10^6^ cells/sample) using Amaxa Nucleofector II (Lonza, Basel, Switzerland) (solution V, program, V-001). After 24 h of siRNA transfection, cell counts were performed using trypan blue, and cells were also collected, the RNA extracted, and RT-qPCR performed as described above. Then, siRNA by Ambion^®^ (life technologies, Carlsbad, CA, USA) was purchased with the sequence as follows (sense strand): *DOHH*, GGCUGUAGCGUGGAAGUUUTT; *HIST1H2AC*, GCGGUGUUAGAGUACCUGATT; *TAF6*, GACUACGCCUUGAA-GCUAATT. Silencer Select Negative Control No. 1 siRNA (Thermo Fisher Scientific; No. 4390843) was used as the negative control. All analyses were performed in biological triplicates.

### 2.7. Statistical Analysis

Statistical analyses were performed using EZR software (version 1.55) (Saitama Medical Center/Jichi Medical University, Saitama, Japan). For the MTS assay after CRISPR activation, a one-way ANOVA with Dunnett’s multiple comparison was used. For all other statistical analyses, an unpaired *t*-test was used. A *p*-value < 0.05 was considered statistically significant for all analyses. Values are expressed as the mean ± standard deviation (SD).

## 3. Results

### 3.1. Identification of NRAS Signaling-Related Growth Factors by Genome-Wide CRISPR Activation Screening

**Figure 1:**
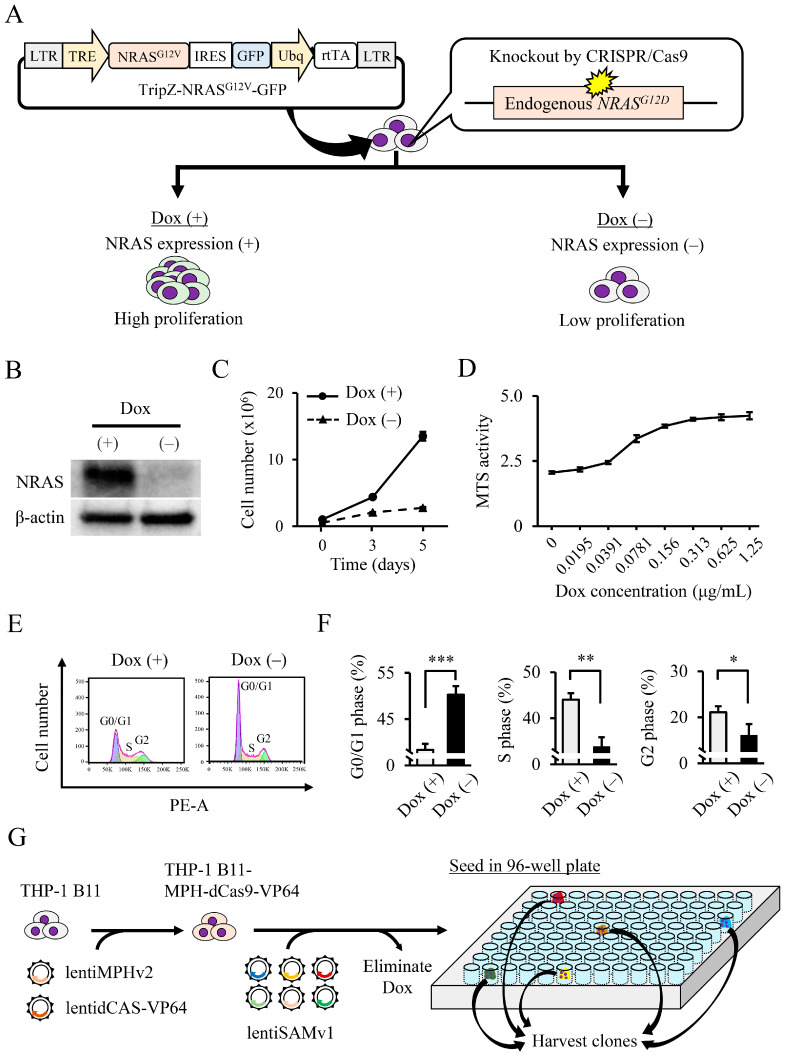
(**A**) Characterization of THP-1 B11 cells. In a previous study, the TripZ-NRAS^G12V^-GFP expression vector was introduced into THP-1 cells, and the endogenous *NRAS^G12D^* gene was knocked out by CRISPR/Cas9. The generated cells (THP-1 B11) were capable of regulating NRAS expression via Dox. With Dox treatment, NRAS was expressed, and the cells showed high proliferation. In the absence of Dox, their cell proliferation was low because they did not express NRAS. (**B**) The induction of NRAS expression in THP-1 B11 cells by the addition of Dox. NRAS expression in THP-1 B11 cells cultured with no Dox or 1 μg/mL of Dox were examined by WB. (**C**) Induction of cell proliferation in THP-1 B11 cells by the addition of Dox. The cell numbers of THP-1 B11 cells cultured with/without Dox were counted at days 3 and 5. Data are expressed as mean ± SD. (**D**) Dox concentration-dependent MTS assay for THP-1 B11 cells. Data are expressed as mean ± SD. (**E**,**F**) Cell cycle analysis: Dox removal caused THP-1 B11 cells to become G0/G1 arrested. (**E**) A representative histogram. Data are expressed as mean ± SD. Statistical significance was determined using an unpaired *t*-test. * *p* < 0.05, ** *p* < 0.01, and *** *p* < 0.001. (**G**) Schematic workflow illustrating genome-wide CRISPR activation screening. The THP-1 B11 cell line capable of Dox-inducible NRAS expression was infected with lentiMPHv2 and lentidCAS-VP64_blast vector, followed by hygromycin and blasticidin selection. The THP-1 B11-MPH-dCas9-VP64 cells were infected with CRISPR activation library (lentiSAMv1) and selected with zeocin, and Dox was removed. We isolated the proliferating cells with no Dox, and then, gRNA sequences integrated into their genomes were analyzed.

The characteristics of THP-1 B11 produced in the previous study are shown in Figure 1A [11]. WB confirmed that NRAS was expressed in the presence of Dox, but the NRAS expression was absent under no Dox (Figure 1B). A decrease in the cell-proliferative capacity after Dox removal is shown in Figure 1C. The MTS assay confirmed that the effect of Dox on the proliferative activity was concentration-dependent (Figure 1D). A G0/G1 arrest was observed with no Dox, while the S and G2 phases decreased significantly (Figure 1E,F). CRISPR activation screening was applied to the THP-1 B11 cells, in which the proliferation activity could be controlled with or without Dox. As shown in Figure 1G, THP-1 B11-MPH-dCas9-VP64 cells constitutively expressing the MS2-P65-HSF1 (MPH) activator helper complex and dCas9-VP64 were generated. After introducing a pooled whole-genome CRISPR activation library into these cells and 2 weeks of drug selection, Dox was removed. The removal of Dox caused many cells to stop proliferating, but some cells proliferated and formed cell colonies. Five such cell colonies were clustered on a 96-well plate and became established (SAM 1–5). After extracting genomic DNA from each of SAM 1–5, an analysis of the integrated gRNA sequences revealed an average of four genes per clone and 21 genes overall (Table 1).

### 3.2. Validation of Candidate Genes Associated with NRAS Proliferation Signaling

**Figure 2:**
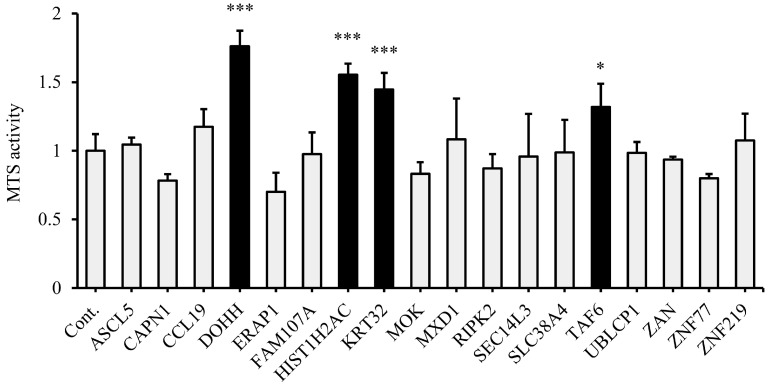
Validation of the candidate genes that induce *NRAS* signaling-associated cell proliferation by MTS assay. MTS assays were performed on cells in which each candidate gene was activated (*n* = 3). Data are presented as mean ± SD. The results compared with control groups were analyzed using one-way ANOVA–Dunnett’s test. * *p* < 0.05 and *** *p* < 0.001.

In order to validate each candidate gene, gRNA expression vectors for each of the 21 candidate genes were constructed and each vector was introduced into THP-1 B11-MPH-dCas9-VP64 cells to establish cells in which only one of each candidate gene was activated by CRISPR activation. Because we were not able to establish clones with gRNAs targeting *CAT*, *DHRS3*, or *KCNJ11*, the remaining 18 candidate genes were evaluated. The cells in which each candidate gene was activated by CRISPR activation were cultured without Dox before determining the cell viability using an MTS assay. The MTS activity of gRNA-transduced cells targeting *DOHH*, *HIST1H2AC*, *KRT32*, or *TAF6* was significantly increased by 1.67-, 1.44-, 1.33-, and 1.29-fold, respectively, compared to that of control cells. (Figure 2).

### 3.3. DOHH, HIST1H2AC, and TAF6 Are Located Downstream of NRAS Signaling

**Figure 3:**
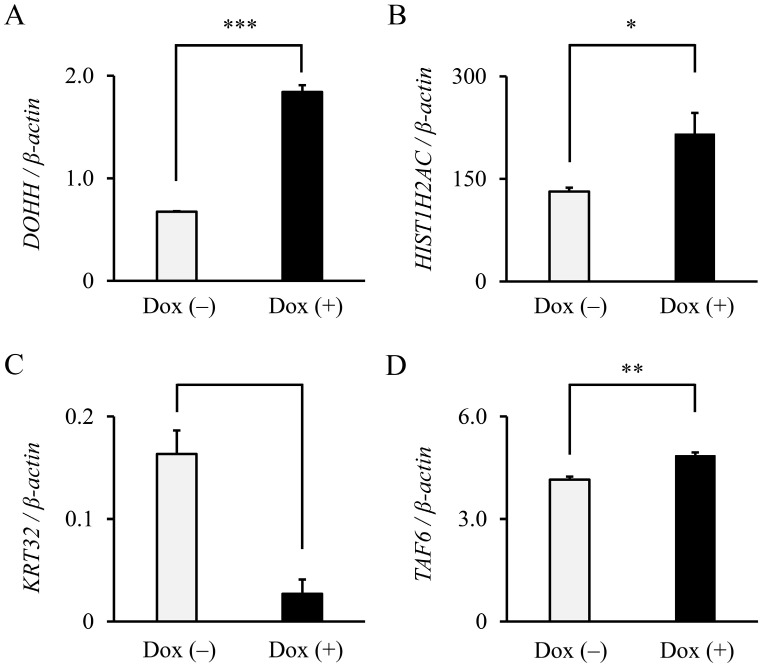
*DOHH*, *HIST1H2AC*, and *TAF6* mRNA were induced by *NRAS* signaling. (**A**) *DOHH*, (**B**) *HIST1H2AC*, (**C**) *KRT32*, and (**D**) *TAF6* mRNA expression in THP-1 B11 cells cultured in the absence and presence of Dox were analyzed by RT-qPCR. Relative mRNA expression was shown as delta–delta Ct values normalized by *β-actin* mRNA (*n* = 3). Data are expressed as mean ± SD. Statistical significance was determined using an unpaired *t*-test. * *p* < 0.05, ** *p* < 0.01, and *** *p* < 0.001.

**Figure 4:**
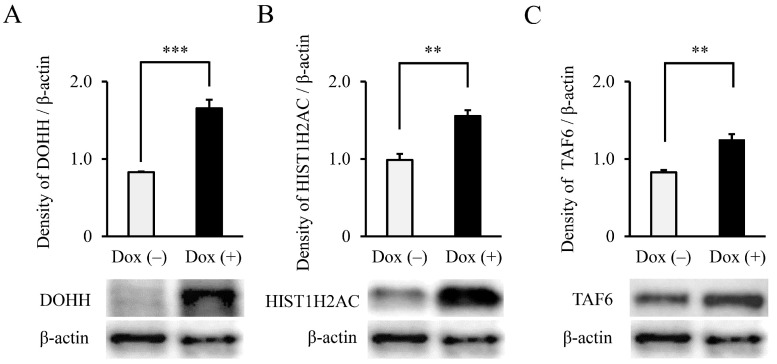
DOHH, HIST1H2AC, and TAF6 proteins induced by *NRAS* signaling. DOHH (**A**), HIST1H2AC (**B**), and TAF6 (**C**) protein expression in THP-1 B11 cells cultured in the absence and presence of Dox were analyzed by WB. Representative blot images are shown in the lower panels. Densitometry values for each protein standardized by β-actin are shown in the upper panels (*n* = 3). Data are expressed as mean ± SD. Statistical significance was determined using an unpaired *t*-test. ** *p* < 0.01, and *** *p* < 0.001. Supplementary File S1 shows the original WB image.

To confirm whether the four identified molecules were indeed regulated by *NRAS^G12V^*, the expression of each candidate gene in the THP-1 B11 cells cultured with and without Dox was analyzed by RT-qPCR. As shown in Figure 3, *DOHH* (2.73-fold, *p* < 0.001), *HIST1H2AC* (1.64-fold, *p* < 0.05), and *TAF6* (1.17-fold, *p* < 0.01) were significantly upregulated through the addition of Dox. In contrast, the *KRT32* expression was significantly decreased in cells with Dox compared with those without Dox. Therefore, we focused on DOHH, HIST1H2AC, and TAF6 as putative candidate molecules located downstream of *NRAS*. NRAS expression was induced by the addition of Dox, resulting in an increased expression of the DOHH, HIST1H2AC, and TAF6 proteins (Figure 4A–C, lower panels). Densitometry showed that the addition of Dox significantly increased the expression of DOHH (2.00-fold, *p* < 0.001), HIST1H2AC (1.57-fold, *p* < 0.01), and TAF6 (1.51-fold, *p* < 0.01) (Figure 4A–C, upper panels), suggesting that DOHH, HIST1H2AC, and TAF6 were transcriptionally induced by NRAS, leading to increased protein levels.

### 3.4. DOHH, HIST1H2AC, and TAF6 Are Located Downstream of ERK Pathway

**Figure 5:**
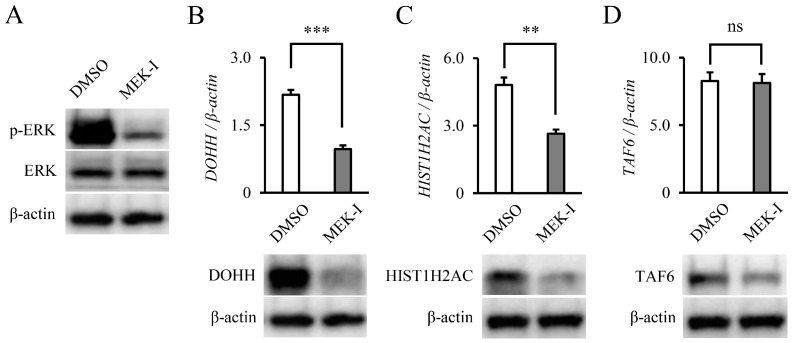
DOHH, HIST1H2AC, and TAF6 are located downstream of ERK. (**A**) After THP-1 parental cell lines had been treated with DMSO or MEK-I (PD184161, 5 μM) for 48 h, ERK and phosphorylated ERK expression were analyzed by WB. (**B**–**D**) Similarly, mRNA expression (**upper** panels) and protein expression (**lower** panels) of DOHH, HIST1H2AC, and TAF6 were analyzed. Data are expressed as mean ± SD. Statistical significance was determined using an unpaired *t*-test. ** *p* < 0.01, and *** *p* < 0.001. ns: no significance.

To confirm whether DOHH, HIST1H2AC, and TAF6 were located downstream of the major *NRAS* signaling pathways, such as the MEK/ERK pathways, the THP-1 parental cell lines were treated with 5 μM MEK inhibitor (PD184161) for 48 h. The decrease in phosphorylated ERK upon MEK inhibition was confirmed by WB (Figure 5A). Under MEK inhibition, the *DOHH* and *HIST1H2AC* mRNA expression was significantly decreased (Figure 5B,C, upper panels). Decreases in the DOHH and HIST1H2AC proteins were also confirmed by WB (Figure 5B,C, lower panels). The TAF6 expression was decreased at the protein level; however, no significant difference was observed in the mRNA expression under MEK inhibition (Figure 5D). The HL-60 cells were treated with 5 μM and the MIA PaCa-2 cells were treated with 10 μM by similar MEK inhibitors for 48h. The expression of only *DOHH* was found to be significantly reduced at the mRNA level in the HL-60 cells (Supplementary Figure S2).

### 3.5. DOHH, HIST1H2AC, and TAF6 Contribute to Leukemia Cell Proliferation

**Figure 6:**
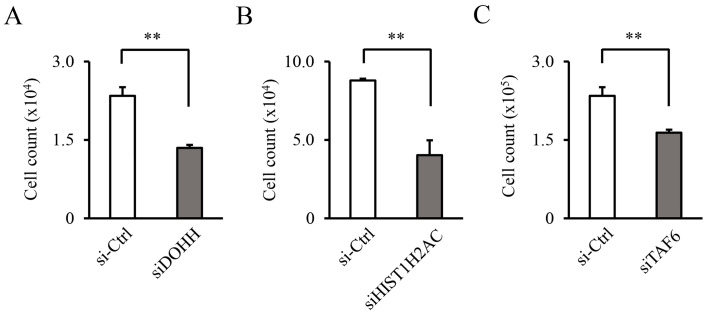
Knockdowns of *DOHH*, *HIST1H2AC*, and *TAF6* by siRNA inhibit cell proliferation. The THP-1 parental cell lines were transfected with siRNAs and the number of viable cells were counted after 48 h. (**A**) *DOHH*, (**B**) *HIST1H2AC*, and (**C**) *TAF6* were targeted and Silencer Select Negative Control No. 1 siRNA (si-Ctrl) was used as a negative control. Data are expressed as mean ± SD. Statistical significance was determined using an unpaired *t*-test. ** *p* < 0.01.

Knockdowns of *DOHH*, *HIST1H2AC*, and *TAF6* by siRNA were performed. The efficiency of the knockdowns against the target genes was confirmed by RT-qPCR (Supplementary Figure S3). The mRNA expression of each gene was significantly downregulated by the corresponding siRNA. The knockdowns of *DOHH*, *HIST1H2AC*, and *TAF6* significantly suppressed cell proliferation (Figure 6).

## 4. Discussion

CRISPR screening can be a powerful tool for identifying unknown regulatory molecules through various innovations, such as reporter genes. In the present study, THP-1 B11 cells, which can switch the expression of *NRAS*, a driver gene essential for proliferation with Dox, were used. [11]. As an example of a study to identify unknown regulatory factors involved in signal transduction, Evron et al. performed CRISPR screening to elucidate Wnt signaling [14]. A vector that expresses a reporter gene in response to Wnt signaling was adapted for CRISPR knockout screening, and a new Wnt signaling repressor, DHX29, was identified.

The RAS/MEK/ERK pathway is a general cancer cell growth signal [15], but treatment with MEK inhibitors downstream of the pathway has been reported to have limited efficacy in some tumors [8]. Therefore, Cai et al. searched for genes that promote the therapeutic effect of MEK-I using *NRAS*-mutant melanoma cells [16]. Comparative analyses of gRNAs with and without MEK-I treatment have confirmed the possibility that PDPK1 is an enhancer of the MEK-I therapeutic effect. In contrast, to search for unknown genes involved in the MEK-I resistance mechanism, Yu et al. applied CRISPR knockout screening to colon cancer cell lines under MEK-I treatment [17]; they focused on the increased expression of resistance-promoting genes in MEK-I-treated cells by transcriptome analysis and identified *GRB7*, a gene that confers resistance to MEK-I.

In the current study, *DOHH*, *HIST1H2AC*, and *TAF6* were identified as *NRAS* proliferation-related genes in the THP-1 B11 cell clones. Deoxyhypsin hydroxylase (DOHH) catalyzes eIF5A activation by performing the hypusination of eukaryotic translation initiation factor 5A (eIF5A) [18]. Because hypusinated eIF5A is involved in the transition from the G1 to S phase of the cell cycle, the suppression of DOHH, which is responsible for its hypusination, leads to cell cycle progression arrest [19]. In prostate cancer cell lines, it has been reported that the microRNA-mediated repression of DOHH expression suppresses the cell-proliferative capacity [20].

TAF6 (TATA-box binding protein associated factor 6) is one of the subunits of the RNA Pol II transcription factor TFIID and plays an essential role in eukaryotic transcription processes [21]. TAF6 is an essential factor in human cell survival because the siRNA-mediated knockdown of *TAF6* causes reduced cell survival [22]. TAF6 has multiple isoforms, and functional differences between each isoform have been reported [23]. In particular, its splicing variant, TAF6δ, has potent apoptotic activity [24,25]. Because the factors involved in apoptosis often act antagonistically between isoforms through selective splicing [26], it is likely that each isoform of TAF6 is tightly regulated at the transcriptional level for cell survival.

HIST1H2AC is one of the histone H2A isoforms and forms three clusters on chromosomes 1 and 6 [27]. In breast cancer cells, HIST1H2AC interacts with the estrogen receptor (ER) and contributes to proliferation by activating the transcription downstream of ER [28]. In the present study, HIST1H2AC was shown to be located downstream of NRAS/ERK, which is consistent with the finding that one of the isoforms, histone H2A isoform type 2-C (HIST2H2AC), is located downstream of the EGFR signaling pathway and contributes to proliferation [29]. Because the incorporation of different histone isoforms into nucleosomes leads to dramatic changes in gene expression [30], HIST1H2AC, which is upregulated downstream of NRAS/ERK, may have important effects on cell growth and survival.

In this study, screening was conducted using THP-1 cell lines depending on *NRAS* signaling; however, the screening method was designed to identify proliferative factors that are not specific to *NRAS*. Therefore, the limitation of this study is that it did not employ a screening method specific to the identification of *NRAS* downstream. The use of multiple *NRAS*-mutant leukemia cells may allow for more *NRAS* mutation-specific screening to elucidate the full extent of *NRAS* signaling.

In the leukemia cell line HL-60 with the *NRAS*^Q61L^ mutation and the pancreatic cancer cell line MIA PaCa-2 with the *KRAS*^G12C^ mutation, the mRNA expression of *DOHH*, *HIST1H2AC*, and *TAF6* were measured with MEK-I treatments. However, only the *DOHH* mRNA expression was lower in the HL-60 cells with the MEK inhibitor treatment, suggesting that *DOHH* may be similarly located downstream of NRAS/ERK in *NRAS*-mutant leukemic HL-60 cells. Interestingly, the expressions of *DOHH*, *HIST1H2AC*, and *TAF6* were upregulated in the MIA PaCa-2 cells with the *KRAS*^G12C^ mutation. The signaling pathway of *NRAS* to the downstream genes *DOHH*, *HIST1H2AC*, and *TAF6* might be dependent on the type of *RAS* mutation and cellular context.

The novel *NRAS* downstream genes identified in the current study may also be potential therapeutic targets for inhibiting the RAS signaling pathway. A detailed functional analysis of DOHH, HIST1H2AC, and TAF6 would lead to the development of new therapeutic targets for *NRAS*-mutant tumors or therapies that are effective in combination with existing downstream NRAS molecular inhibitors, such as MEK inhibitors.

## 5. Conclusions

CRISPR activation screening identified three novel *NRAS* downstream genes: *DOHH*, *HIST1H2AC*, and *TAF6*. These genes contribute to the proliferation of the leukemia cell THP-1 and may be new therapeutic targets for *NRAS*-mutant leukemia cells.

## Figures and Tables

**Table 1:** Gene candidates for potentially compensable *NRAS* signaling.

Clone	Gene Name	Sequence of gRNA
SAM-1	*CAT*	GGCCAAGATTGGAAGCCCAA
	*CCL19*	CCCTGTTGCCCCCCTCTTTC
	*DOHH*	TTTTATTCCTCACCTACTTA
	*SLC38A4*	AAAAGACGCAATTTACCAAC
SAM-2	*ASCL5*	TAGGGCGATGGCTCTTGTTA
	*CAPN1*	CGGAAGGGCACCGCGGGGAA
	*DHRS3*	TTTTTTTCTGGAGACGGGGT
	*KCNJ11*	TCCGGATCTCTCCCACTAAC
	*MXD1*	TTGCGAATCCTGTCACCAGT
	*TAF6*	GTTTCCCTGCCTCCGTTTTG
SAM-3	*ERAP1*	TCGGTCCCCAACTTGAGCAC
	*FAM107A*	TGAAGTTCCAATGACATTCA
	*HIST1H2AC*	TTGTCTTCCAATTAACTAAG
	*KRT32*	ATTTGGCTAAAGCAGGAGTC
	*SEC14L3*	TCTGTCCCCAAGCCAAGCAG
	*ZNF219*	ACTCCTTCCCTGGTATGTCC
SAM-4	*MOK*	AAGGCTATCGTCCACGTAGT
	*RIPK2*	TGGGACGGGCGGCTGGGAAG
	*ZAN*	GGACTGCAAACGGCTGGACG
SAM-5	*UBLCP1*	TGTTCCGAATGAAGCTTAAA
	*ZNF77*	CCGCCCCTGCCTGTCCTGAT

## Data Availability

Not applicable.

## Supplementary Materials

The following supporting information can be downloaded at: https://www.mdpi.com/article/10.3390/biology11111551/s1, Figure S1: Analysis of DOHH, HIST1H2AC, and TAF6 expression in THP-1 B11 cells following MEK inhibitor treatment. Figure S2: *DOHH*, *HIST1H2AC*, and *TAF6* expression with MEK-I treatment in HL-60 cell lines harboring *NRAS*^Q61L^ mutation and MIA PaCa-2 cell lines harboring *KRAS**^G12C^* mutations. Figure S3: Suppression of mRNA expression by siRNA knockdown was confirmed by RT-qPCR. File S1: Original images of Western blot analysis.
