# Supplementary material for: Identification of NRAS Downstream Genes with CRISPR Activation Screening
Source: Biology (Basel). 2022 Oct 23;11(11):1551. doi: 10.3390/biology11111551 (PMC9687188; doi:10.3390/biology11111551)
Supplement: Supplementary file 1 [file biology-11-01551-s001.zip › biology-1904269-supplementary.pdf]

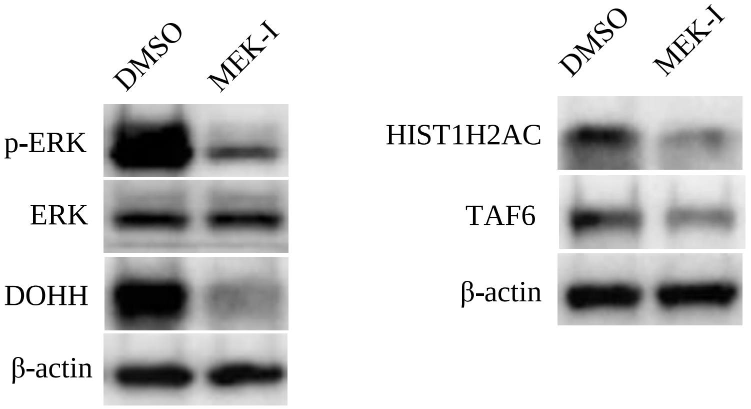

Figure S1. Analysis of DOHH, HIST1H2AC, and TAF6 expression in THP-1 B11 cells following MEK inhibitor treatment. Expression of ERK, phosphorylated ERK, DOHH, HIST1H2AC, and TAF6 were analyzed by WB for THP-1 parental cell lines treated with DMSO or MEK-I. The two blot images were performed at different time points using the same cells.

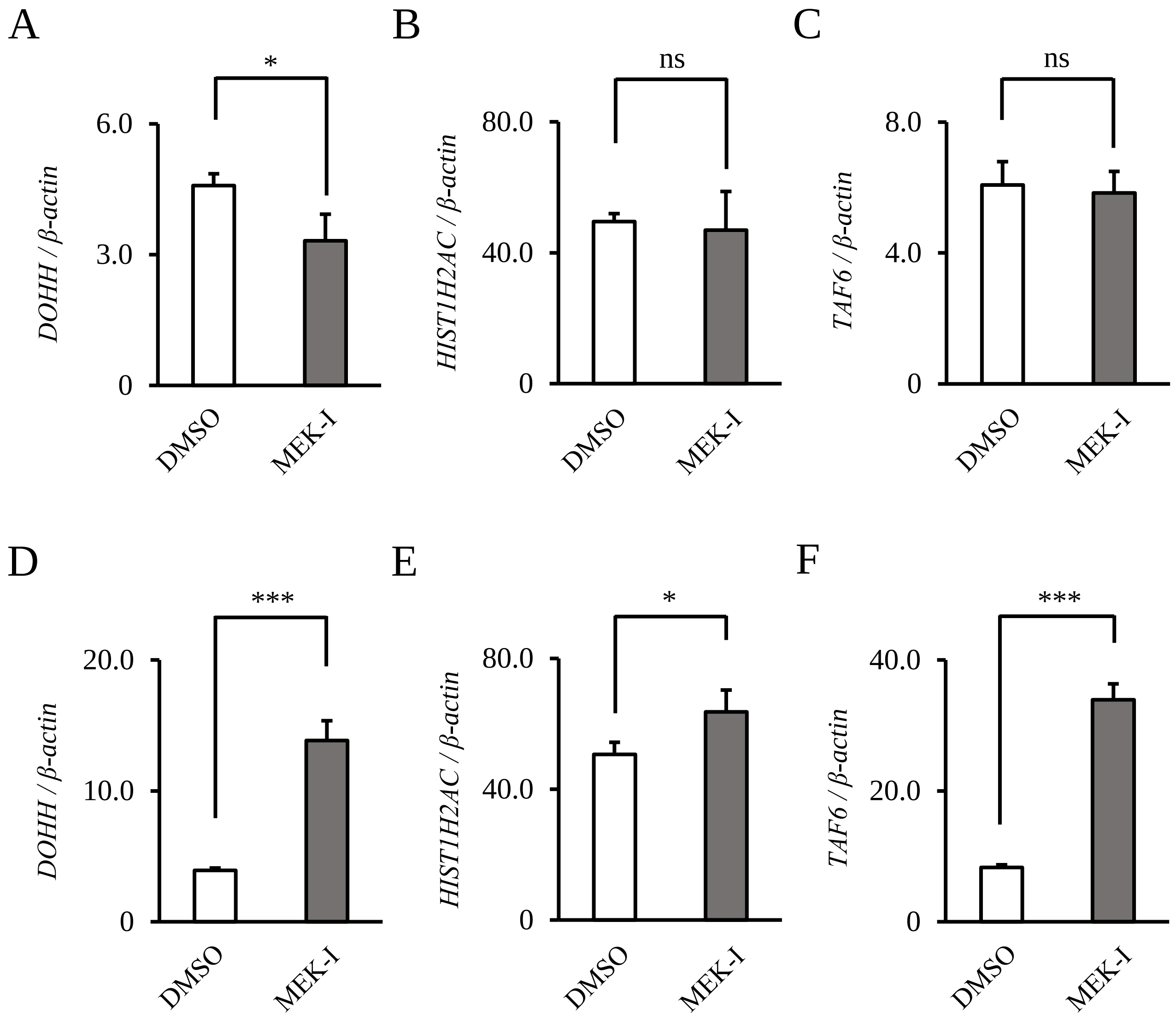

Figure S2. DOHH, HIST1H2AC, and TAF6 expression with MEK-I treatment in HL-60 cell lines harboring NRAS^{Q61L} mutation and MIA PaCa-2 harboring KRAS^{G12C} mutations. (A–C) HL-60 cells were treated with MEK-I (PD184161, 5 μM) for 48 h. RT-qPCR was analyzed after reverse transcription of RNA extracted from collected cells. Relative mRNA expression of *DOHH*, *HIST1H2AC*, and *TAF6* was shown as delta-delta Ct values normalized by *β-actin* mRNA (n=3). (D–F) MIA PaCa-2 were treated with MEK-I (PD184161, 10 μM) for 48h and similarly analyzed by RT-qPCR (n=3). Data are expressed as mean±SD. Statistical significance was determined using an unpaired *t*-test. *$p < 0.05$, **$p < 0.01$, ***$p < 0.001$. ns: no significance.

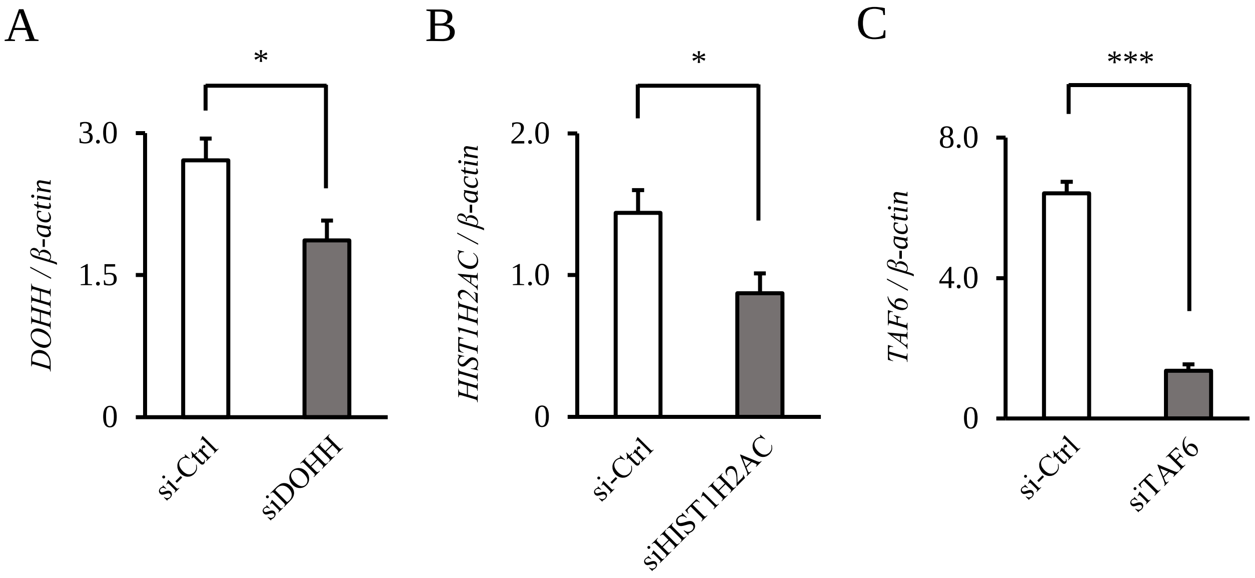

Figure S3. Suppression of mRNA expression by siRNA knockdown was confirmed by RT-qPCR. The THP-1 parental cell lines (1.0×10^6 cells/sample) were transfected with siRNA using Amaxa Nucleofector II (solution V, program V-001), and the cells were retrieved after 24 h. siRNAs targeting different genes in *DOHH* (A), *HIST1H2AC* (B), and *TAF6* (C) were used. RT-qPCR was analyzed after reverse transcription of RNA extracted from collected cells. Relative mRNA expression was shown as delta-delta Ct values normalized by *β-actin* mRNA (n=3). si-Ctrl was used as a negative control. Data are expressed as mean±SD. Statistical significance was determined using an unpaired *t*-test. *$p < 0.05$, **$p < 0.01$, ***$p < 0.001$.
