# Supplementary material for: Identification of NRAS Downstream Genes with CRISPR Activation Screening
Source: Biology (Basel). 2022 Oct 23;11(11):1551. doi: 10.3390/biology11111551 (PMC9687188; doi:10.3390/biology11111551)

# Original data for Figure 1B

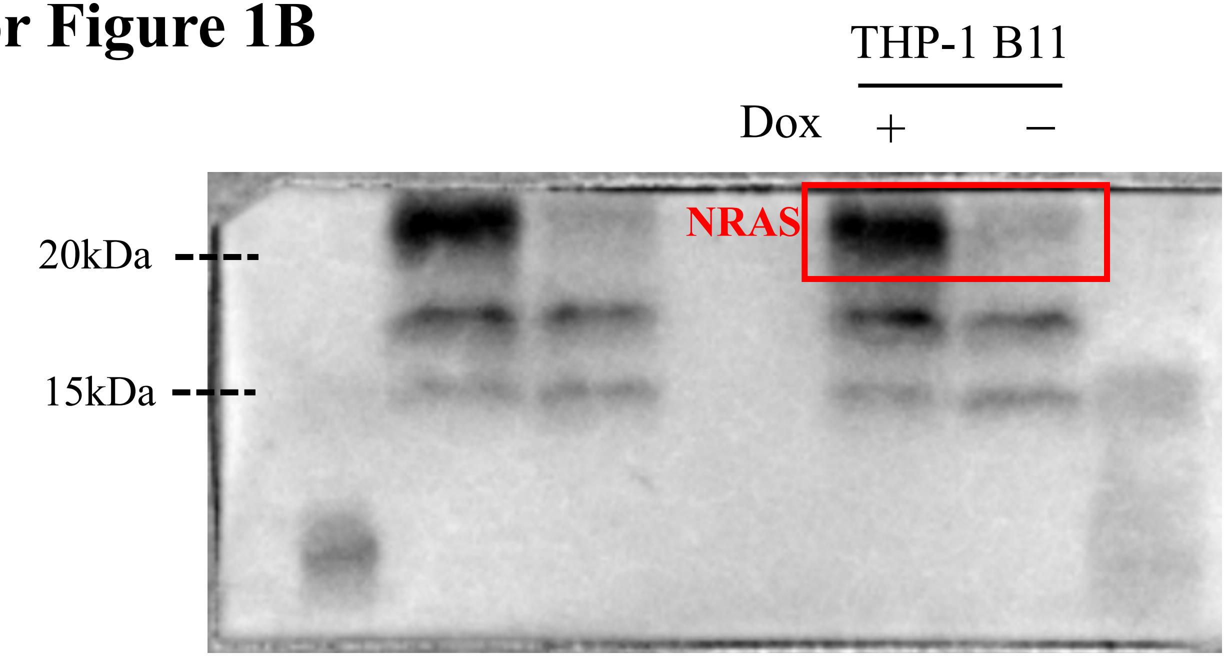


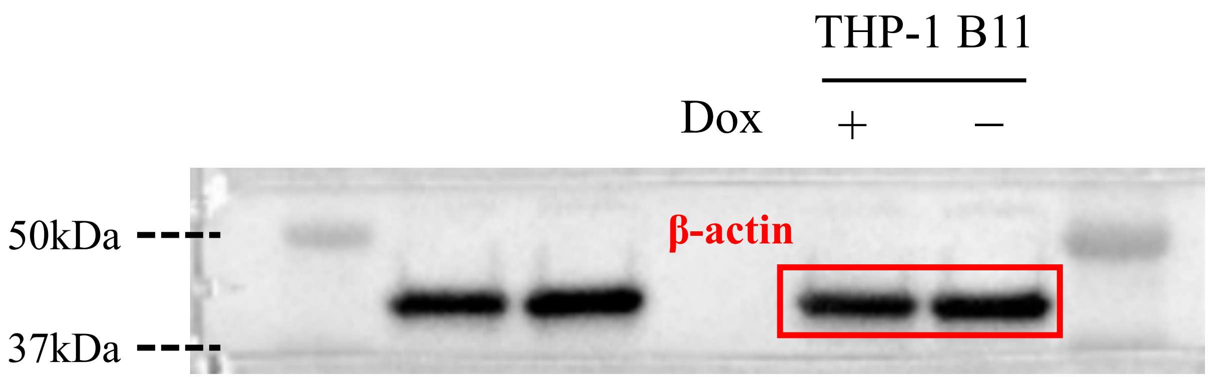

# Original raw data for Figure 4

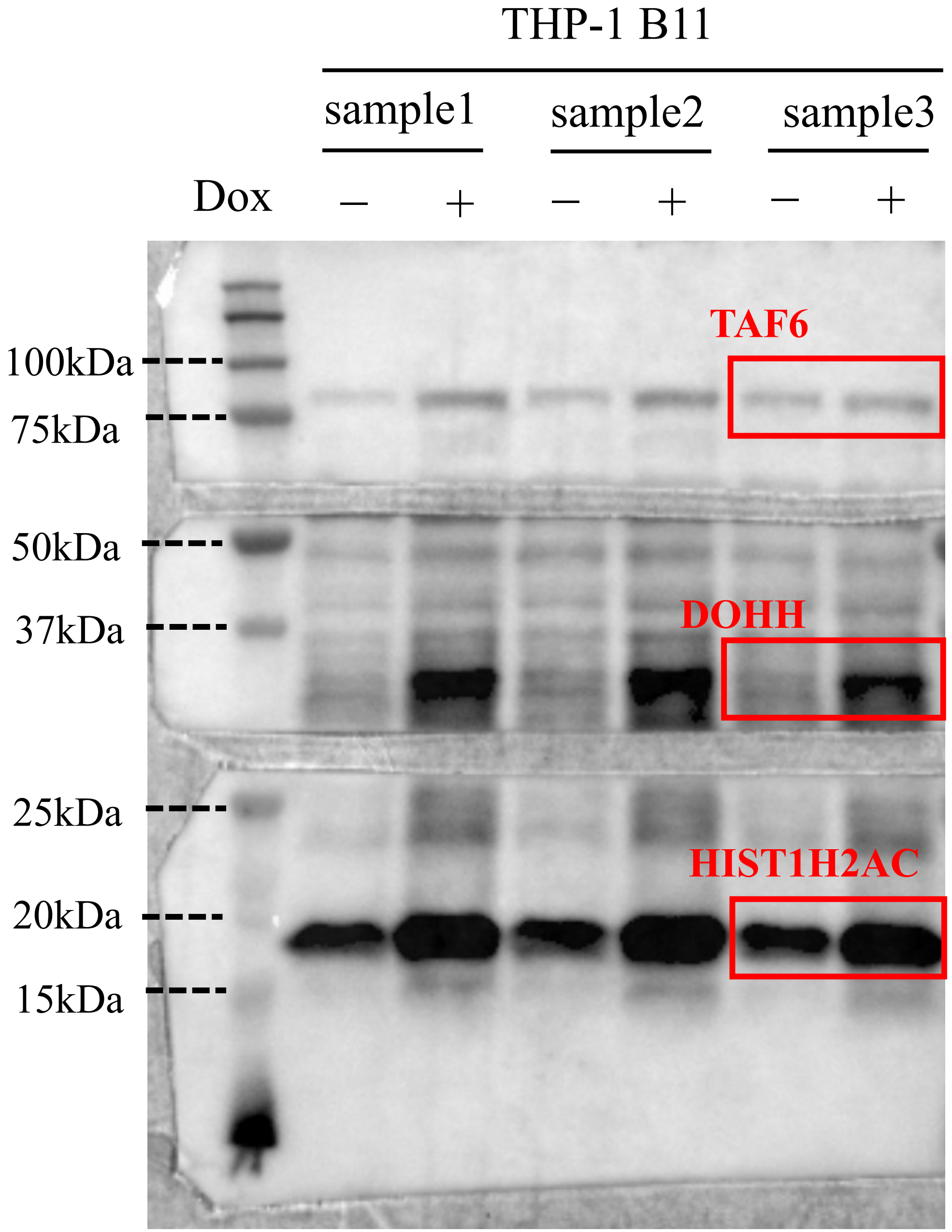


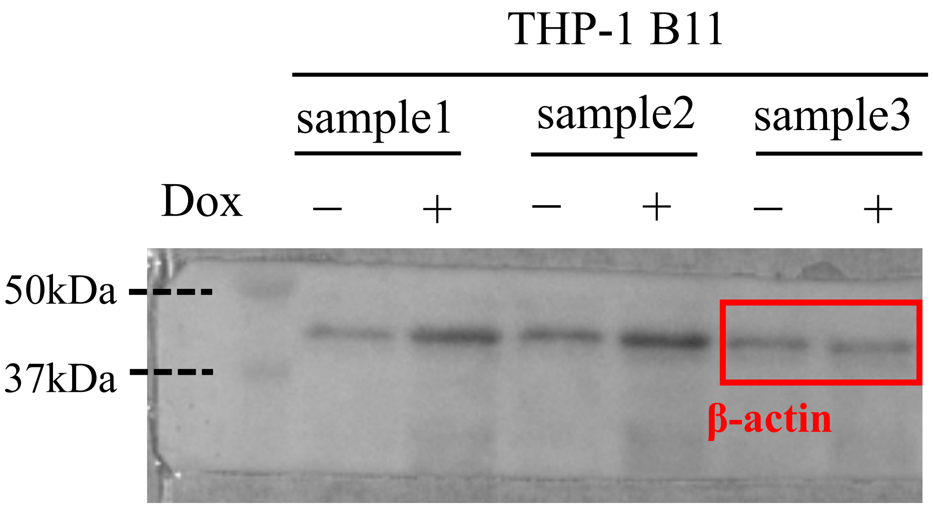

# Original raw data for Figure 5①

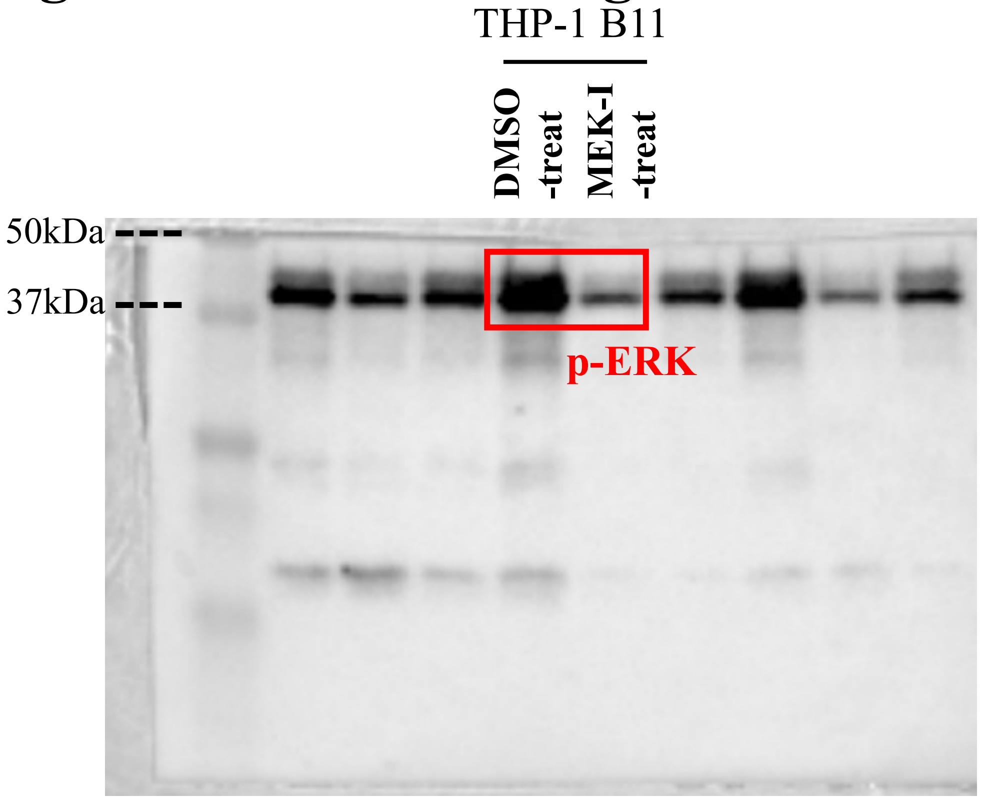


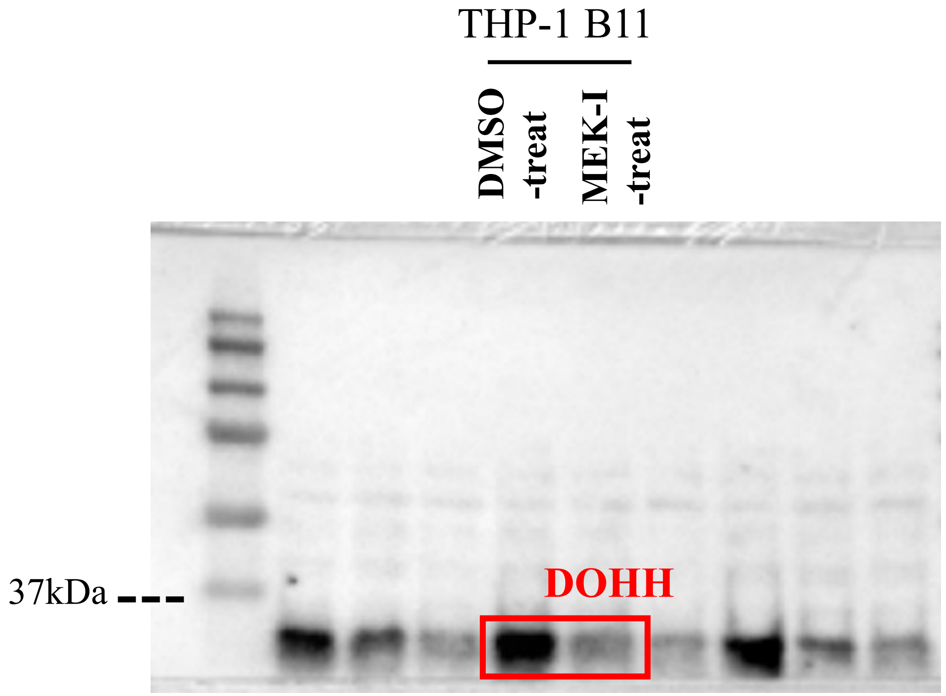


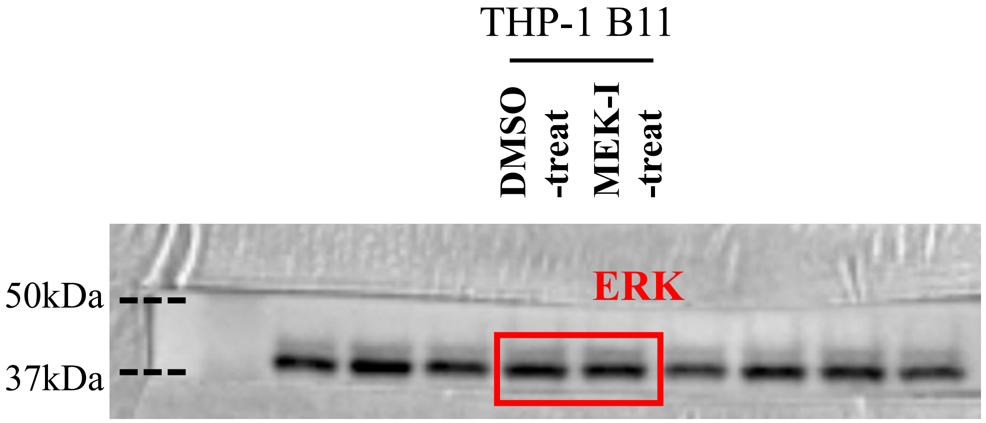


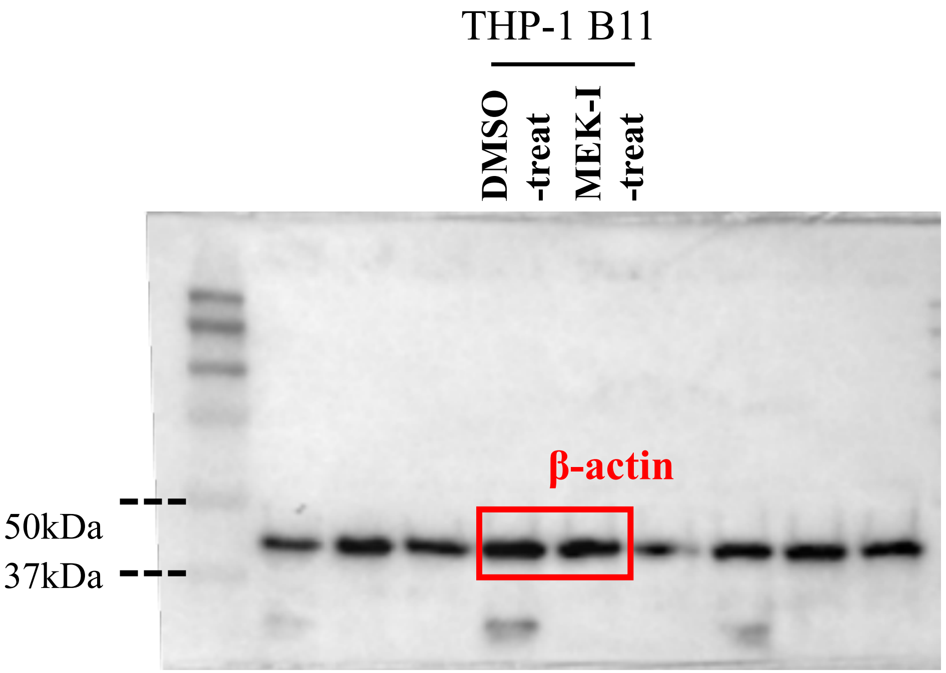

# Original raw data for Figure 5②

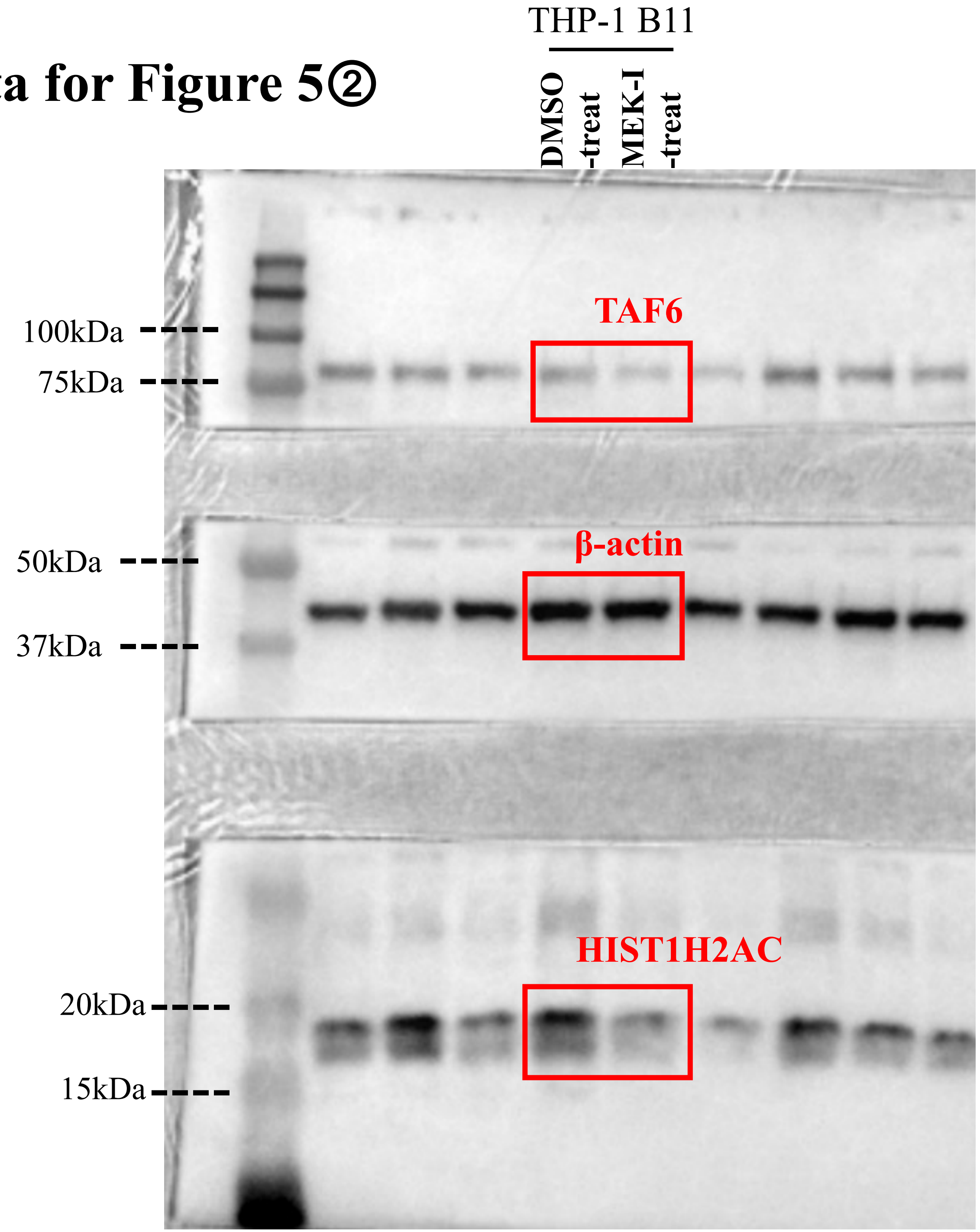

Supplement: Supplementary file 1 [file biology-11-01551-s001.zip › File S1 Original raw data for WB.PDF]
